# ^1^H, ^13^C, and ^15^N resonance assignment of the C terminal region of the disordered postsynaptic scaffold protein GKAP

**DOI:** 10.1007/s12104-025-10253-2

**Published:** 2025-11-05

**Authors:** Eszter Nagy-Kanta, Anna Sánta, Zsófia E. Kálmán, Jessica Amy Li, Perttu Permi, Zoltán Gáspári, Bálint Péterfia

**Affiliations:** 1 Faculty of Information Technology and Bionincs, Pázmány Péter Catholic University, Budapest, Hungary; 2 Department of Biological and Environmental Science, University of Jyväskylä, Jyväskylä, Finland; 3 Department of Chemistry, University of Jyväskylä, Jyväskylä, Finland; 4 Institute of Biotechnology, Helsinki Institute of Life Science, University of Helsinki, Helsinki, Finland

**Keywords:** Intrinsically disordered protein, Postsynaptic density, GKAP, Shank1, PDZ domain, Helical propensity

## Abstract

Guanylate kinase-associated protein, GKAP, is a largely disordered scaffold protein with multiple interaction partners, playing a role in the organization of the postsynaptic protein network. Its C-terminus contains a binding motif for the PDZ domain of Shank proteins, another class of postsynaptic scaffolds. Based on predictions, this motif is preceded by a ~ 40-residue disordered segment with no known additional binding sites or established functional role. Here we report the expression, purification, and ^1^H, ^13^C and ^15^N resonance assignment of the GKAP Ct43 construct containing the C-terminal 43 residues. This region is functionally intact, having a binding affinity to Shank1 PDZ in the micromolar range. Chemical shifts indicate that this region is indeed disordered but displays helical propensity in two regions. The short, slightly helical segment immediately before a PDZ-binding motif is somewhat comparable to the structural organization observed at the C-terminus of the E6 and RSK1 proteins, but its significance in GKAP is yet to be explored.

**Supplementary Information:**

The online version contains supplementary material available at 10.1007/s12104-025-10253-2.

## Biological context

The postsynaptic density (PSD), an elaborate and dynamic network beneath the postsynaptic membrane, is responsible for the modulation of signal processing in excitatory synapses, and it is believed to be a key contributor to the development of several cognitive disorders, such as ASD or schizophrenia. On the one hand, the PSD is highly structured both vertically and horizontally. It has a layered architecture through its depth, linking the transmembrane receptors to the cytoskeleton, and also forms so-called nanodomains that group receptors together at specific membrane regions (Droogers and MacGillavry 2023). On the other hand, the PSD is both variable between cell types and brain regions, and highly dynamic as it can undergo reorganization as a response to neural activity, as well as upon the circadian cycle etc. (Cizeron et al. 2020; Droogers and MacGillavry 2023; Grant 2019; Zhu et al. 2018).

**Fig. 1:**
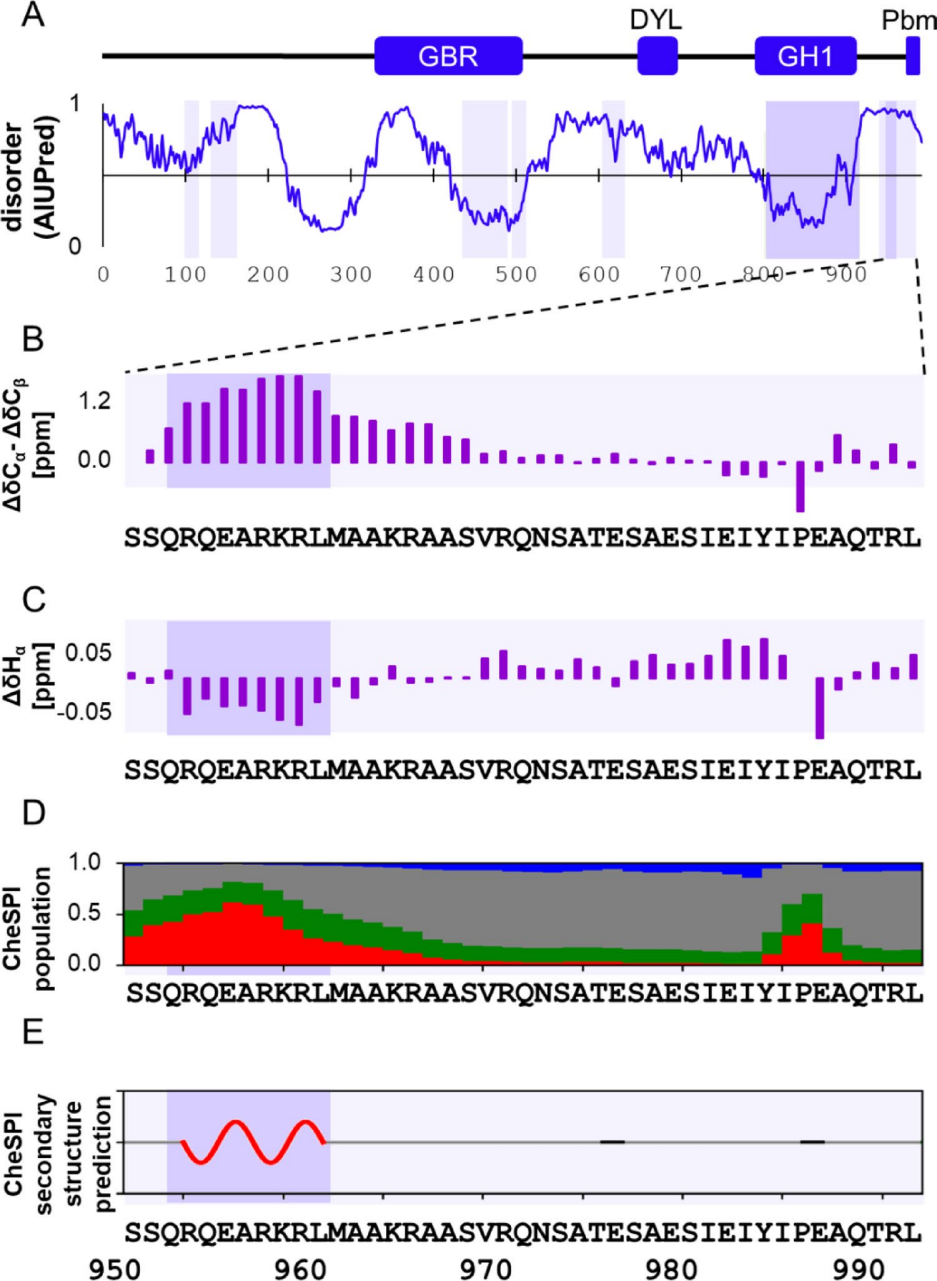
(**a**) rGKAP1a (UniProt ID: P97836) disorder prediction (known domains and regions: GBR: GK binding region, DYL: dynein light chain binding region, GH1: guanylate homology domain 1, Pbm: PDZ binding motif). Helical regions predicted by AlphaFold2 are highlighted with light purple background, experimentally proven helical regions are highlighted with darker purple background (by (Tong et al. 2014), PDB:4Y0R and by us in this current study); (**b**) C_α_-C_β_ secondary chemical shifts (calculated with POTENCI); (**c**) H_α_ secondary chemical shifts (calculated with POTENCI); (**d**) Stacked bar plot of CheSPI populations of “extended” (blue), “helical” (red), “turn” (green), and “non-folded” (grey) structural elements; (**e**) Cartoon representation of the most confidently predicted secondary structure by CheSPI. In (**b**)-(**e**) helical regions predicted by AlphaFold2 are highlighted with light purple background, experimentally proven helical regions are highlighted with darker purple background (by Tong et al., PDB:4Y0R and by us in this current study)

The PSD is organized by large scaffold proteins, a significant portion of which harbor long intrinsically disordered regions. One of the most extreme examples is GKAP (Guanylate kinase associated protein), a protein of almost 1000 residues, predicted to be practically entirely disordered (see Fig. 1a). There is very limited experimental information on the structure of GKAP; only one longer segment, its GH1 domain, near the C-terminus, has been crystallized as an MBP fusion construct (PDB: 4R0Y, (Tong et al. 2014). We have recently characterized the LC8-binding region of rat GKAP spanning residues 655–711 and proved that this segment is intrinsically disordered. NMR titration with the dimeric LC8 hub protein indicated that this region even retains a substantial degree of flexibility in its bound state (Nagy-Kanta et al. 2025). Similar behavior was observed for several other multivalent LC8-binding proteins (Clark et al. 2018; Walker et al. 2023). This observation is eventually in line with the generally expected role of disorder in scaffold proteins (Cortese et al. 2008), and might be key during the dynamic reorganization of the PSD. Our NMR investigations also revealed possible interactions by residues flanking the strictly defined, “core” LC8 binding motifs, with different patterns for the two binding sites (Nagy-Kanta et al. 2025).

The C-terminal region of GKAP can interact with the PDZ domain of Shank proteins, another family of large postsynaptic scaffolds exhibiting substantial intrinsic disorder. PDZ domains are abundant domains mediating protein: protein interactions (Ivarsson 2012). They have a well-characterized 3D structure, containing both helical and extended secondary structural elements. Ligands bind to PDZ domains via beta augmentation, with SLiMs (Short Linear Motifs). Although the archetypal PDZ ligand is a C-terminal peptide, there are also internal segments that can bind to PDZ domains. The Shank1 PDZ has been described to interact with a plethora of partners, both C-terminal and internal ones. The consensus sequence for C-terminal Shank1-binding motifs is x-T-x-(L/F)–COOH, whereas for internal motifs it is exclusively x-T-x-F-x, where x is any amino acid (Ali et al. 2021). The crystal structure of the Shank1:GKAP complex is available, where Shank1 PDZ is a dimer, but it only contains a peptide corresponding to the 6 C-terminal residues of GKAP (PDB:1Q3P, (Im et al. 2003). Another crystal structure, 5OVC, contains a 7-residue GKAP segment in complex with the Shank3 PDZ, here Shank3 PDZ is in monomeric form (Ponna et al. 2018). However, PDZ dimerization was also observed in another crystallization experiment by Zeng et al. (PDB:5IZU (Zeng et al. 2016), where a longer segment of Shank3 was complexed with a longer, 15 residue-long C terminus of SAPAP3, a GKAP-related protein. The SAPAP3 peptide used is almost identical to the C terminus of GKAP (with just one alteration in position − 13, from D to E). In this case, not only the last 6 amino acids but the whole of the 15 residue-long segment participates in beta augmentation, moreover, it also facilitates dimerization between two distant beta sheets of the Shank chains (Zeng et al. 2016).

Although the primary specificity of peptide: PDZ interactions is widely considered to be confined to a small segment, namely, the C-terminal 4 residues, from p_0_ to p_−4_, there are indications that the wider sequence context, definitely up to p_−10_, may also play a role (Luck et al. 2012), as it is also clear from the PDB:5IZU structure (Zeng et al. 2016). There are other studies hinting at the functional contribution of flanking residues for a diverse set of other domain: peptide interactions (Chica et al. 2009; Nagy-Kanta et al. 2025; Palopoli et al. 2018; Simonsen et al. 2025), indicating the role of positions as far as 10–15, or even 20 residues from the ‘core’ motif. For PDZ domains, such interactions can be deduced for some ligands from the available NMR structures of PDZ: peptide complexes (PDB: 2M3M, 2KOH, 2K20, (Mischo et al. 2013; Tyler et al. 2010). Of these, the C-terminal segment of HPV51 oncoprotein E6 has been studied by NMR, using a longer construct incorporating a Zn-binding domain before the PDZ-binding C-terminus (PDB: 2M3L). This domain adopts a compact fold, including a C-terminal helix stabilized by a bidentate H-bond formed by the guanidino group of Arg to the side chain oxygens of Gln and Ser (Mischo et al. 2013). This helix is immediately before the C-terminal ~ 7 residues that are largely disordered and form the primary PDZ-binding region.

The C-terminal PDZ binding motif of the RSK1 kinase is preceded by a long, ~ 40-residue disordered region also harboring an autoinhibitory site and a MAPK-binding site. The region before the PDZ-interacting residues was shown to have helical propensity in solution. In contrast to E6, no additional interaction with the PDZ2 of the MAGI1 protein was observed beyond the 6 C-terminal residues, neither in the crystal structure (PDB:5N5F (He et al. 2019) nor with NMR spectroscopy, in spite of using a long, 49-residue C-terminal RSK1 construct (Gógl et al. 2018).

In contrast to the E6 protein but somewhat similar to RSK1, GKAP is predicted to possess an extended disordered C-terminal region of ~ 40 residues. We are not aware of any other predicted binding sites in this region, and the amino acid content here differs from that of RSK1. Some crystal structures of Shank1 as well as the analogous Shank3 indicated dimerization of the PDZ while bound to GKAP or other partners (PDB:1Q3P, 5IZU, 3QJN, 3L4F, 7A9B, (Ali et al. 2021; Im et al. 2003, 2010; Lee et al. 2011; Zeng et al. 2016) whereas other experiments, including our own NMR results, strongly suggested that Shank1 PDZ is monomeric in solution (PDB:5OVC (Ponna et al. 2018; Sánta et al. 2022). These seemingly contradictory results call for a more detailed analysis of the Shank1:GKAP interaction and the resulting complexes. In this respect, the exact functional significance of the long disordered C-terminal segment in GKAP, preceding the C-terminal PDZ binding motif, is worth investigating in detail, and, to our current knowledge, there is no structural information available on this region. Based on AlphaFold2 structure prediction, the already described GH1 domain (PDB:4R0Y (Tong et al. 2014) is not the only region that might harbor alpha-helical features (see Fig. 1a).

In this study, we describe the expression, purification and resonance assignment of the C-terminal 43 residues of GKAP, a construct termed Ct43 hereafter. This segment was chosen because currently there is no atomic-level experimental data available for it, and is deemed sufficiently long to be suitable for the investigation of potential interactions outside the primary binding site in future studies.

## Methods and experiments

### Cloning, expression and purification

We chose to investigate the C terminal 43 residues (Ct43) of the *Rattus norvegicus* GKAP protein (UniProt P97836, residues 950–992, 100% identical to the corresponding region of the human homolog DLGAP1, Uniprot O14490, residues 935–977). The GKAP-Ct43 construct incorporates the C terminal PDZ binding motif EAQTRL and 37 additional residues as an extended flanking region, expected to be largely disordered. The construct also contains an N-terminal 4-residue GSHM sequence remaining from TEV protease cleavage of the 6xHis-tag.

The inserts were amplified from the rGKAP cDNA template sequence, kindly provided by Enora Moutin, using the following forward and reverse primers: 5’-AAAAACATATGAGCTCACAGCGCCAGGA and 5’-AAAAACTCGAGCTAgaggcgggtctgcg, respectively. After digestion with restriction enzymes NdeI and XhoI, the inserts were ligated into a modified pET-15b vector (Novagen) containing a 6xHis-tag and tobacco etch virus (TEV) cleavage site coding sequence, Lac operon and Ampicillin resistance gene. The sequence of the plasmid vector construct was verified with Sanger sequencing.

Protein expression was induced from this plasmid vector in BL21 (DE3) *E. coli* cells (Novagen) with 1 mM of IPTG (Isopropyl β-D-1- thiogalactopyranoside, Sigma) at 5 MFU cell density, in 250 ml liquid culture. For unlabeled protein production LB medium, while for isotopically labeled samples M9 minimal medium was used. The M9 medium was prepared freshly (composed of 22 mM KH_2_PO_4_, 50 mM Na_2_HPO_4_, 8.5 mM NaCl, 2 mM MgSO_4_, vitamin mix and trace metal mix (Azatian et al. 2019), supplemented with 0.25% ^15^NH_4_Cl (Cambridge Isotope Laboratories, Cambridge, MA) and 0.4% ^13^C-D-glucose. Protein expression was performed overnight at 20 °C, then cells were harvested, centrifuged and stored at −20 °C until further use.

Cells were extracted with ultrasonic homogenization in lysis buffer (300 mM NaCl, 50 mM NaPi, pH 7.4; 10% cell suspension). Cell debris was sedimented by centrifugation (11 000 rpm, 30 min, 4 °C), and the supernatant was affinity purified with a 5 ml Bio-Scale Mini Nuvia™ IMAC Ni-affinity column (Bio-Rad), followed by overnight His-tag cleavage at 10 °C with 0.1 mg/ml TEV protease. After concentration with an Amicon^®^ Ultra Centrifugal Filter tube, the sample was further purified with size exclusion chromatography using a Superdex ^®^ 200 increase 10/300 GL column (Cytiva). The recombinant protein was eluted in a low salt, low pH NaPi buffer (50 mM NaPi, 20 mM NaCl, pH 6.0), then was lyophilized for safe transport. Molecular weight and sample purity was validated with SDS-PAGE and LC-MS, and protein concentration was estimated based on absorbance measurement with Nanodrop.

For the biolayer interferometry measurements, the TEV cleavage step was omitted, so the 6xHis tag on the GKAP-Ct43 constructions could have been utilized for loading molecules on the biosensors.

Shank1-PDZ (UniProt: Q9WV48, residues 654–768, 100% identical with the human homolog UniProt: Q9Y566, residues 654–768) production and purification was also performed according to the protocol described in detail earlier (Sánta et al. 2022). Reverse IMAC purification was applied with Nuvia™ IMAC Ni-affinity beads after TEV cleavage to eliminate all the 6xHis tag peptides and the uncleaved protein molecules from the solution (causing nonspecific binding).

### NMR spectroscopy and chemical shift assignment

NMR experiments were performed at the University of Jyväskylä. Spectra were acquired using 0.3 mM ^15^N, ^13^C -labeled GKAP-Ct43 samples in 2/98% D_2_O/H_2_O at pH 6.0. Chemical shifts were referenced to internal 2,2,-dimethyl-2-silapentane-5-sulfonic acid (DSS). All data were acquired at 298.15 K on a Bruker AVANCE III HD 800 MHz spectrometer, equipped with a TCI ^1^H/^13^C/^15^N Z-gradient cryoprobe. Data was collected and processed with TopSpin version 3.5 pl7.

The following experiments were used in the resonance assignment of GKAP-Ct43: ^1^H-^15^N HSQC, ^1^H-^13^C HSQC-aliphatic, ^1^H-^13^C HSQC-aromatic, HNCACB, HN(CO)CACB, HNCO, i(HCA)CO(CA)NH (Mäntylahti et al. 2009), HBHA(CO)NH, and, to assign the amide nitrogen of the proline residue in the construct, 4D HACANCOi (Karjalainen et al. 2020). For chemical shift assignment, CCPNmr Analysis v3.1 software was used.

Sequential neighborhood-, temperature- and pH-corrected random coil chemical shifts, and from that secondary chemical shift values were calculated with POTENCI (Nielsen and Mulder 2018). CheSPI (Nielsen and Mulder 2021) analysis was performed based on the C’, C_α_, C_β_, N and H_N_ chemical shifts.

### Biolayer interferometry

Biolayer interferometry kinetic measurements were performed on a BLItz instrument (Fortebio, USA) using Sartorius NTA biosensors. In the course of the kinetic measurements protein and buffer samples were administered to the sensors in 250 μl volume in black walled, 0.5 ml tubes. GKAP-Ct43 protein molecules were immobilized to the NTA sensors as baits by their His-tag in 200 nM concentration, and Shank1-PDZ was associated to them as ligand in 4-, 2-, 1- and 0.5 μm concentrations.

The protocol of the kinetic measurements was composed of a 30 s baseline and 120 s loading steps, followed by a 30 s baseline, a 60 s association and a 60 s dissociation step. The composition of the kinetic buffer was 50 mM NaPi, pH 7.4, 20 mM of NaCl, 20 mM Imidazole, 0.1% of BSA and 0.02% of Tween20. Buffers and bait and ligand solutions were equilibrated to room temperature before measurements. It is important to note that a difference of 0–3 °C in temperature could occur between different measurements, since temperature control of the BLItz instrument is not possible. We applied control measurements: (1) we added kinetic buffer instead of bait at the loading step, then measured association with Shank1-PDZ, to see nonspecific binding of Shank to the sensor; (2) we applied kinetic buffer during the association phase (with no Shank-PDZ protein) to have a background measurement. During data evaluation, the background plot was subtracted from the measured plots. Dissociation constant (K_d_) was calculated as average from three independent measurements, each determined from the BLI kinetic curves using the global fitting method of the data analysis software.

### Extent of assignments and data deposition

We confirmed that the binding affinity to Shank1-PDZ of the GKAP-Ct43 protein is 3.1 ± 0.7 µM (for one representative interferogram see Supplementary Fig. 1).

**Fig. 2:**
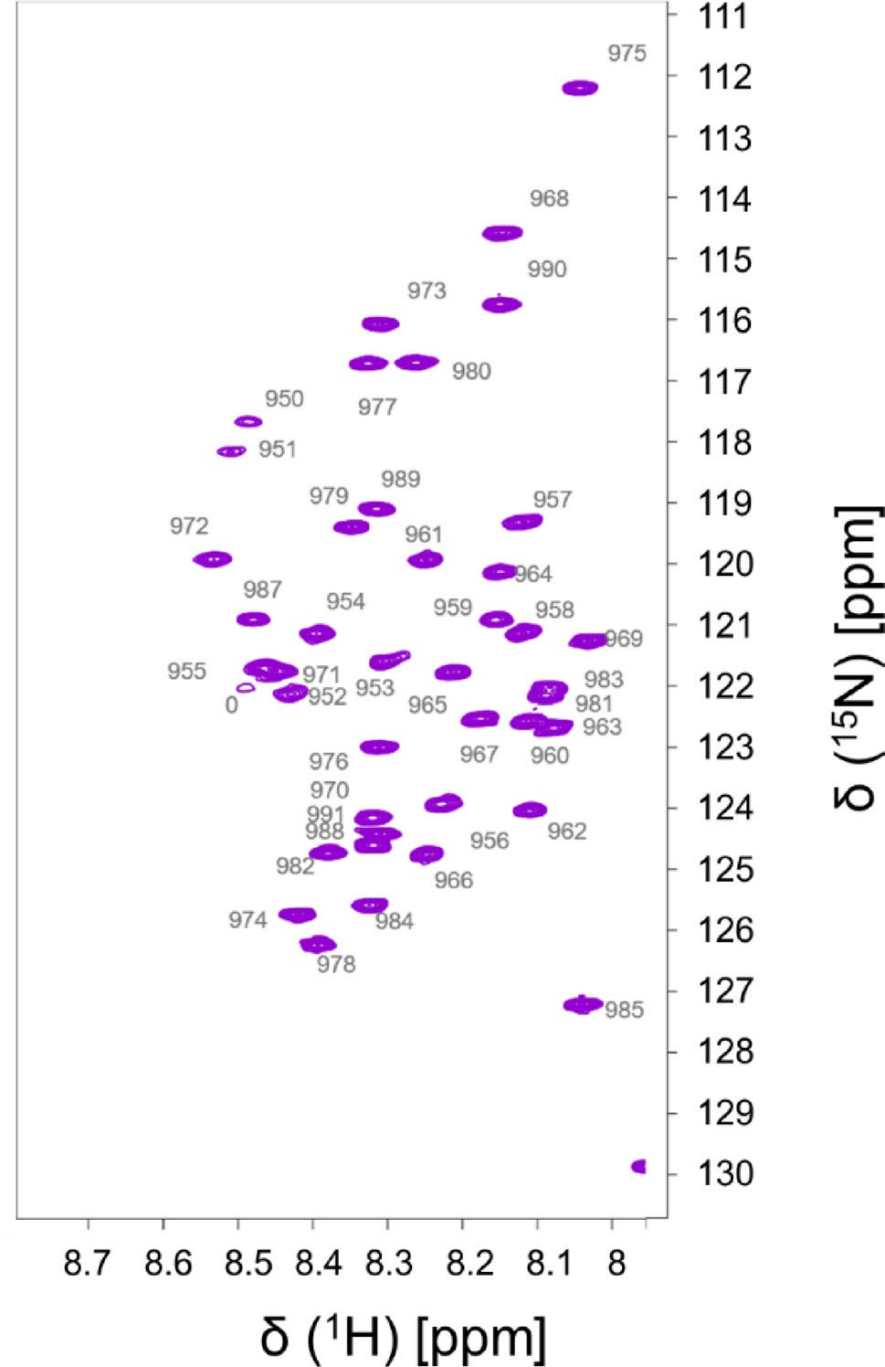
^1^H^15^N HSQC spectrum of GKAP-Ct43

Complete backbone (N, H, C’, C_α_ and H_α_) chemical shift assignment as well as the identification of all C_β_ and H_β_ atom resonances was carried out for the whole GKAP-Ct43 segment. Chemical shifts were deposited to BMRB under the accession number 53245. Signal dispersion in the ^1^H, ^15^N HSQC spectrum of the GKAP-Ct43 construct is ~ 0.6 ppm in the ^1^H, and ~ 18 ppm in the ^15^N dimension (Fig. 2).

Sequential neighborhood-, temperature- and pH-corrected secondary chemical shift values calculated with POTENCI (Nielsen and Mulder 2018) are between − 1.12 and + 2 ppm for C_α_-C_β_ chemical shifts and between − 0.12 and + 0.08 ppm for H_α_ atom chemical shifts (Fig. 1b, c).

The analysis of chemical shifts indicates that the construct is intrinsically disordered along its full length. The calculated difference between C_ɑ_ and C_β_ secondary chemical shifts indicate a slight helical preference between positions 950–967. CheSPI analysis (Nielsen and Mulder 2021) also shows a mainly disordered structure with no preferences for stable secondary structure formation along the C terminal segment immediately before the PDZ-binding motif (residues 971–992), but with definitive helical propensity in the extended flanking region, between residues 950–970 (Fig. 1d, e).

We note the presence of a unique pattern of secondary chemical shifts for the Ile985-Pro986 residue pair (Fig. 1b, c). While we have no explicit rationalization for the relatively large negative secondary C_ɑ_-C_β_ shift difference of Ile985, and the large negative H_ɑ_ secondary chemical shift of Pro986, we suspect the effect of the steric constraints induced by the asymmetrically branching Ile side chain and the pyrrolidine ring. Our current data do not enable the unambiguous determination whether Pro986 adopts cis or trans structure but we have not observed any signs of heterogeneity that might arise from isomerization.

The helical propensity visible in Fig. 1e is significant but smaller than the one for highly helical segments like for example the Drebrin SAH (single alpha helix) region, where secondary H_ɑ_ shifts are almost twice as large in absolute value (Varga et al. 2025). The presence of a helical segment approximately matches the AlphaFold2 predictions, although the observed helix is shorter than the predicted one towards the C-terminus. The difference at the N-terminal region of the helix might be attributed to the fact that the construct starts after the N-terminal residue of the AlphaFold2-predicted helix. Based on secondary structure prediction, this segment was identified as helix 4 in the study of the GH1 X-ray structure. Although not visible in the deposited structural model (PDB: 4R0Y), a weak interaction between a peptide corresponding to this helix (residues 950–971) and helices 1–3 (807–911) was demonstrated by ITC measurements (Tong et al. 2014). Our results confirm the conformational preferences of this region in solution.

The even smaller helical preferences, most likely the presence of a single turn, for residues YIPE between positions 984–987, immediately before the PDZ-binding motif seems to be harder to interpret (Fig. 1d, e). We note that the position of this turn roughly coincides with the location of the C-terminal helix of the Zn-binding domain of the E6 oncoprotein (Mischo et al. 2013) and the helical propensity observed in RSK1 (Gógl et al. 2018). The exact relevance of this segment in the GKAP-PDZ interaction will be explored in future studies.

## Supplementary Information

Below is the link to the electronic supplementary material.

Supplementary Material 1

## Data Availability

The assigned chemical shifts have been deposited in the BioMagResBank under ID 53245.

## References

[CR1] Ali M, McAuley MM, Lüchow S, Knapp S, Joerger AC, Ivarsson Y (2021) Integrated analysis of Shank1 PDZ interactions with C-terminal and internal binding motifs. Curr Res Struct Biol 3:41–50. 10.1016/j.crstbi.2021.01.001 34235485 10.1016/j.crstbi.2021.01.001 PMC8244488

[CR2] Azatian SB, Kaur N, Latham MP (2019) Increasing the buffering capacity of minimal media leads to higher protein yield. J Biomol NMR 73(1–2):11–17. 10.1007/s10858-018-00222-4 30613903 10.1007/s10858-018-00222-4 PMC6441617

[CR3] Chica C, Diella F, Gibson TJ (2009) Evidence for the concerted evolution between short linear protein motifs and their flanking regions. PLoS ONE 4(7):e6052. 10.1371/journal.pone.0006052 19584925 10.1371/journal.pone.0006052 PMC2702822

[CR4] Cizeron M, Qiu Z, Koniaris B, Gokhale R, Komiyama NH, Fransén E, Grant SG N (2020) A brainwide atlas of synapses across the mouse life span. Science 369(6501):270–275. 10.1126/science.aba3163 32527927 10.1126/science.aba3163 PMC7115813

[CR5] Clark S, Myers JB, King A, Fiala R, Novacek J, Pearce G, Heierhorst J, Reichow SL, Barbar EJ (2018) Multivalency regulates activity in an intrinsically disordered transcription factor. Elife. 10.7554/eLife.36258 10.7554/eLife.36258 PMC5963919 29714690

[CR6] Cortese MS, Uversky VN, Dunker AK (2008) Intrinsic disorder in scaffold proteins: getting more from less. Prog Biophys Mol Biol 98(1):85–106. 10.1016/j.pbiomolbio.2008.05.007 18619997 10.1016/j.pbiomolbio.2008.05.007 PMC2671330

[CR7] Droogers WJ, MacGillavry HD (2023) Plasticity of postsynaptic nanostructure. Mol Cell Neurosci 124:103819. 10.1016/j.mcn.2023.103819 36720293 10.1016/j.mcn.2023.103819

[CR8] Gógl G, Biri-Kovács B, Póti ÁL, Vadászi H, Szeder B, Bodor A, Schlosser G, Ács A, Turiák L, Buday L, Alexa A, Nyitray L, Reményi A (2018) Dynamic control of RSK complexes by phosphoswitch-based regulation. FEBS J 285(1):46–71. 10.1111/febs.14311 29083550 10.1111/febs.14311

[CR9] Grant SGN (2019) Synapse diversity and synaptome architecture in human genetic disorders. Hum Mol Genet 28(R2):R219–R225. 10.1093/hmg/ddz178 31348488 10.1093/hmg/ddz178 PMC6872429

[CR10] He D, Piergentili C, Ross J, Tarrant E, Tuck LR, Mackay CL, McIver Z, Waldron KJ, Clarke DJ, Marles-Wright J (2019) Conservation of the structural and functional architecture of encapsulated ferritins in bacteria and archaea. Biochem J 476(6):975–989. 10.1042/BCJ20180922 30837306 10.1042/BCJ20180922

[CR11] Im YJ, Lee JH, Park SH, Park SJ, Rho S-H, Kang GB, Kim E, Eom SH (2003) Crystal structure of the Shank PDZ-ligand complex reveals a class I PDZ interaction and a novel PDZ-PDZ dimerization. J Biol Chem 278(48):48099–48104. 10.1074/jbc.M306919200 12954649 10.1074/jbc.M306919200

[CR12] Im YJ, Kang GB, Lee JH, Park KR, Song HE, Kim E, Song WK, Park D, Eom SH (2010) Structural basis for asymmetric association of the betapix coiled coil and Shank PDZ. J Mol Biol 397(2):457–466. 10.1016/j.jmb.2010.01.048 20117114 10.1016/j.jmb.2010.01.048

[CR13] Ivarsson Y (2012) Plasticity of PDZ domains in ligand recognition and signaling. FEBS Lett 586(17):2638–2647. 10.1016/j.febslet.2012.04.015 22576124 10.1016/j.febslet.2012.04.015 PMC7094393

[CR14] Karjalainen M, Tossavainen H, Hellman M, Permi P (2020) HACANCOi: a new Hα-detected experiment for backbone resonance assignment of intrinsically disordered proteins. J Biomol NMR 74(12):741–752. 10.1007/s10858-020-00347-5 33118136 10.1007/s10858-020-00347-5 PMC7701164

[CR15] Lee JH, Park H, Park SJ, Kim HJ, Eom SH (2011) The structural flexibility of the shank1 PDZ domain is important for its binding to different ligands. Biochem Biophys Res Commun 407(1):207–212. 10.1016/j.bbrc.2011.02.141 21376703 10.1016/j.bbrc.2011.02.141

[CR16] Luck K, Charbonnier S, Travé G (2012) The emerging contribution of sequence context to the specificity of protein interactions mediated by PDZ domains. FEBS Lett 586(17):2648–2661. 10.1016/j.febslet.2012.03.056 22709956 10.1016/j.febslet.2012.03.056

[CR17] Mäntylahti S, Tossavainen H, Hellman M, Permi P (2009) An intraresidual i(HCA)CO(CA)NH experiment for the assignment of main-chain resonances in 15 N, 13 C labeled proteins. J Biomol NMR 45(3):301–310. 10.1007/s10858-009-9373-4 19768387 10.1007/s10858-009-9373-4

[CR18] Mischo A, Ohlenschläger O, Hortschansky P, Ramachandran R, Görlach M (2013) Structural insights into a wildtype domain of the oncoprotein E6 and its interaction with a PDZ domain. PLoS ONE 8(4):e62584. 10.1371/journal.pone.0062584 23638119 10.1371/journal.pone.0062584 PMC3640046

[CR19] Nagy-Kanta E, Kálmán ZE, Tossavainen H, Juhász T, Farkas F, Hegedüs J, Keresztes M, Beke-Somfai T, Gáspári Z, Permi P, Péterfia B (2025) Residual flexibility in the topologically constrained multivalent complex between the GKAP scaffold and LC8 hub proteins. FEBS J. 10.1111/febs.70219 10.1111/febs.70219 PMC12797016 40843979

[CR20] Nielsen JT, Mulder FAA (2018) POTENCI: prediction of temperature, neighbor and pH-corrected chemical shifts for intrinsically disordered proteins. J Biomol NMR 70(3):141–165. 10.1007/s10858-018-0166-5 29399725 10.1007/s10858-018-0166-5

[CR21] Nielsen JT, Mulder FAA (2021) CheSPI: chemical shift secondary structure population inference. J Biomol NMR 75(6–7):273–291. 10.1007/s10858-021-00374-w 34146207 10.1007/s10858-021-00374-w

[CR22] Palopoli N, González Foutel NS, Gibson TJ, Chemes LB (2018) Short linear motif core and flanking regions modulate retinoblastoma protein binding affinity and specificity. Protein Eng Des Sel 31(3):69–77. 10.1093/protein/gzx068 29370437 10.1093/protein/gzx068

[CR23] Ponna SK, Ruskamo S, Myllykoski M, Keller C, Boeckers TM, Kursula P (2018) Structural basis for PDZ domain interactions in the post-synaptic density scaffolding protein Shank3. J Neurochem 145(6):449–463. 10.1111/jnc.14322 29473168 10.1111/jnc.14322

[CR24] Sánta A, Czajlik A, Batta G, Péterfia B, Gáspári Z (2022) Resonance assignment of the Shank1 PDZ domain. Biomol NMR Assign 16(1):121–127. 10.1007/s12104-022-10069-4 35083656 10.1007/s12104-022-10069-4 PMC9068651

[CR25] Simonsen S, Larsen FB, Søgaard CK, Jonsson N, Lindorff-Larsen K, Bruheim P, Otterlei M, Hartmann-Petersen R, Kragelund BB (2025) Extreme multivalency and a composite short linear motif facilitate PCNA-binding, localisation and abundance of p21 (CDKN1A). FEBS J. 10.1111/febs.70133 10.1111/febs.70133 PMC12366268 40392971

[CR26] Tong J, Yang H, Eom SH, Chun C, Im YJ (2014) Structure of the GH1 domain of guanylate kinase-associated protein from *rattus norvegicus*. Biochem Biophys Res Commun 452(1):130–135. 10.1016/j.bbrc.2014.08.073 25152391 10.1016/j.bbrc.2014.08.073

[CR27] Tyler RC, Peterson FC, Volkman BF (2010) Distal interactions within the par3-VE-cadherin complex. Biochemistry 49(5):951–957. 10.1021/bi9017335 20047332 10.1021/bi9017335 PMC2819025

[CR28] Varga S, Péterfia BF, Dudola D, Farkas V, Jeffries CM, Permi P, Gáspári Z (2025) Dynamic interchange of local residue-residue interactions in the largely extended single alpha-helix in drebrin. Biochem J 482(8):383–399. 10.1042/BCJ20253036 40192062 10.1042/BCJ20253036 PMC12203971

[CR29] Walker DR, Jara KA, Rolland AD, Brooks C, Hare W, Swansiger AK, Reardon PN, Prell JS, Barbar EJ (2023) Linker length drives heterogeneity of multivalent complexes of hub protein LC8 and transcription factor ASCIZ. Biomolecules. 10.3390/biom13030404 10.3390/biom13030404 PMC10046861 36979339

[CR30] Zeng M, Shang Y, Guo T, He Q, Yung W-H, Liu K, Zhang M (2016) A binding site outside the canonical PDZ domain determines the specific interaction between Shank and SAPAP and their function. Proc Natl Acad Sci U S A 113(22):E3081–E3090. 10.1073/pnas.1523265113 27185935 10.1073/pnas.1523265113 PMC4896714

[CR31] Zhu F, Cizeron M, Qiu Z, Benavides-Piccione R, Kopanitsa MV, Skene NG, Koniaris B, DeFelipe J, Fransén E, Komiyama NH, Grant SG (2018) Architecture of the mouse brain synaptome. Neuron 99(4):781-799e10. 10.1016/j.neuron.2018.07.007 30078578 10.1016/j.neuron.2018.07.007 PMC6117470

