# Supplementary material for: 1H, 13C, and 15N resonance assignment of the C terminal region of the disordered postsynaptic scaffold protein GKAP
Source: Biomol NMR Assign. 2025 Nov 5;20(1):2. doi: 10.1007/s12104-025-10253-2 (PMC12589307; doi:10.1007/s12104-025-10253-2)
Supplement: Supplementary file 1 — Supplementary Material 1 [file 12104_2025_10253_MOESM1_ESM.docx]

[**^Biomolecular NMR Assignments^**](https://link.springer.com/journal/12104)

**^1^H, ^13^C, and ^15^N resonance assignment of the C terminal region of the disordered postsynaptic scaffold protein GKAP**

Eszter Nagy-Kanta^1^, Anna Sánta^1^, Zsófia E. Kálmán^1^, Jessica Amy Li^1^, Perttu Permi^2,3,4^, Zoltán Gáspári^1^, Bálint Péterfia^1^

^1^ Pázmány Péter Catholic University, Faculty of Information Technology and Bionincs, Budapest, Hungary

^2^ Department of Biological and Environmental Science, University of Jyväskylä, Jyväskylä, Finland

^3^ Department of Chemistry, University of Jyväskylä, Jyväskylä, Finland

^4^ Institute of Biotechnology, Helsinki Institute of Life Science, University of Helsinki, Helsinki, Finland

**Corresponding author contact details**

Bálint Péterfia

Práter utca 50/a, 1083, Budapest, Hungary

+36-1-886-4739

[peterfia.balint.ferenc@itk.ppke.hu](mailto:peterfia.balint.ferenc@itk.ppke.hu)

**Supplementary Figure 1**


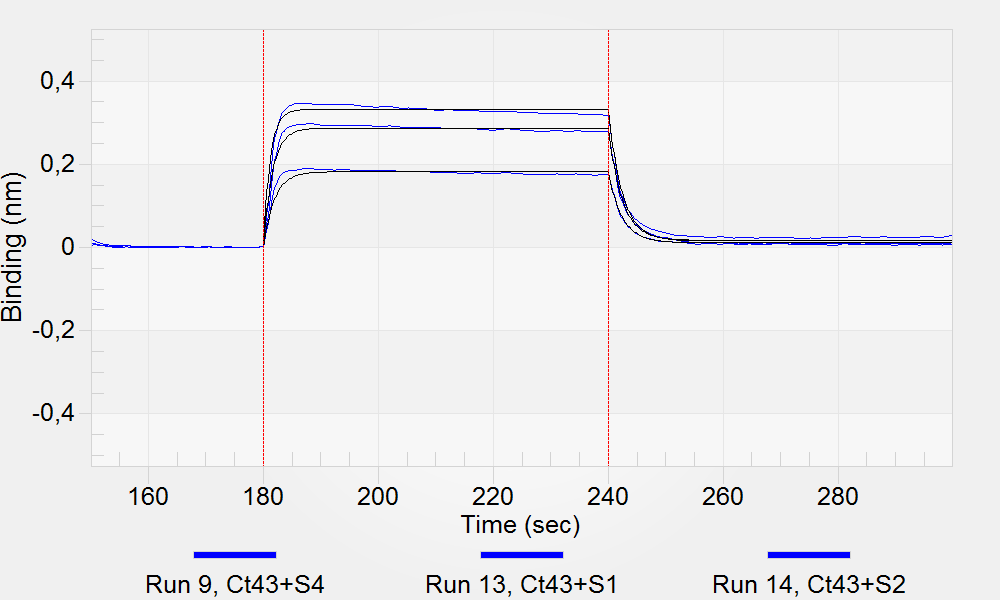


***Suppl. Fig. 1*** *Biolayer interferometry (BLI) results of Shank1-PDZ domain against GKAP-Ct43. One representative interferogram was chosen from three independent measurements. Association and dissociation curves are represented in colored lines according to the analyte (Shank1-PDZ) concentration („S4” meaning 4 μM, „S2” 2 μM, „S1” 1 μM) and their fitting curves are represented in black lines. The resulting K_d_ value is 3.1 ± 0.7 μM*
